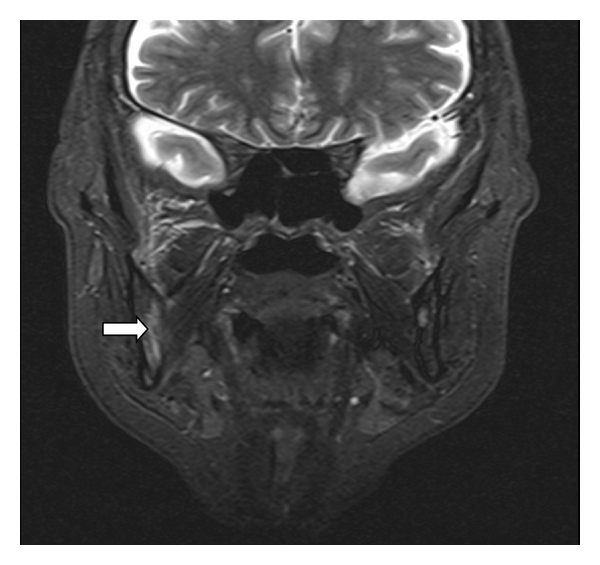# Primary Extranodal, Extralymphatic Hodgkin Lymphoma of the Mandible

**DOI:** 10.1155/2011/387570

**Published:** 2011-06-23

**Authors:** Guido Ricardo Gonzalez-Fontal, Joaquin D. Rosales, Roberto Jaramillo, Andres F. Henao-Martinez

**Affiliations:** ^1^Department of Internal Medicine and Division of Hematology-Oncology, Department of Pathology, CES University-Fundación Valle del Lili, Carrera 98 # 18-49, Cali, Colombia; ^2^Division of Infectious Diseases, University of Colorado Denver, 12700 E 19th Avenue, Aurora, CO 80045, USA

## Abstract

Primary extranodal, extralymphatic Hodgkin lymphomas (PEEHLs) are a rare occurrence. When they are encountered, they become diagnostic challenges. We are describing the uniqueness of a case of PEEHL affecting the mandible with his early response to the available chemotherapy.

## 1. Case

A 68-year-old hispanic male presented with an oral ulcer 1 month after an orthodontic procedure. His complains were mainly pain at the right inner side of the mouth and adjacent ipsilateral facial swelling. There was no fever, weight loss, or other constitutional symptoms referred at presentation. The lesion and facial edema progressively increased over the last 16 months since the initial presentation. Five biopsies and ancillary diagnostic tests were unremarkable. Past history was only positive for cholecystectomy, knee and cataract surgery. He was then hospitalized for further workup.

**Figure 1:**
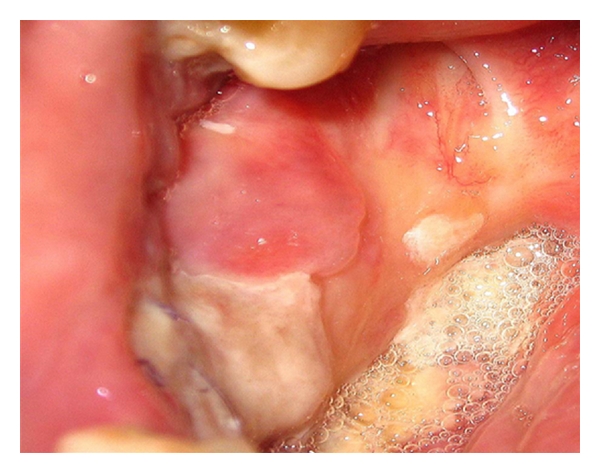


On arrival, his vital signs were within normal limits, body mass index of 29. The physical examination was only relevant for the presence of an oral ulcer involving the buccal mucosa and extending to the edentulous alveolar ridge in the right posterior mandibular vestibule (Figure 1). 

**Figure 2:**
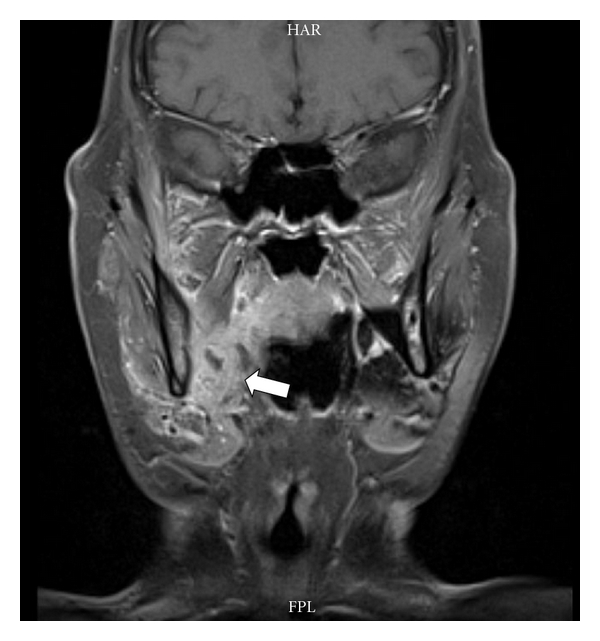


**Figure 3:**
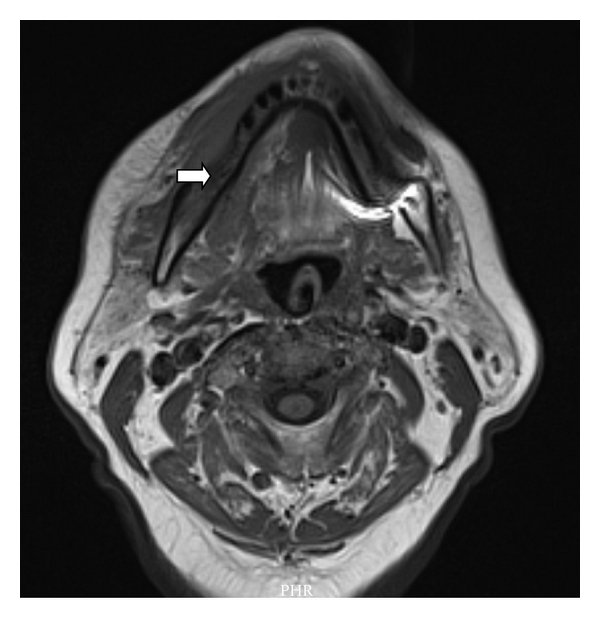


**Figure 4:**
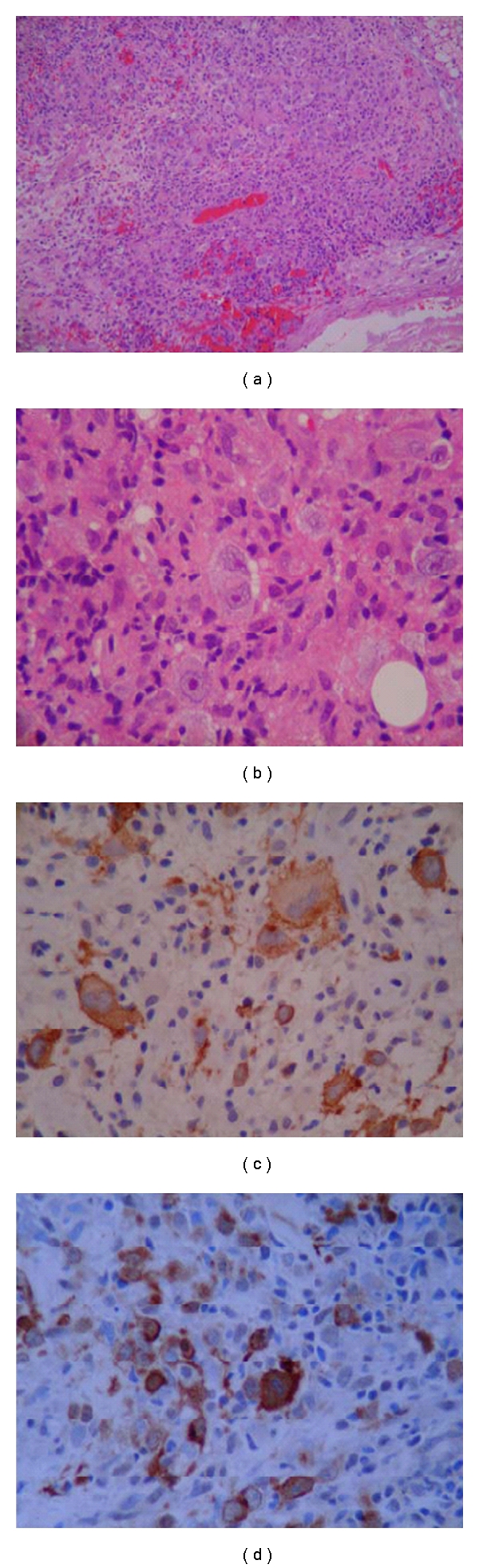


Swelling over the nasogenial sulcus was also noted. There was no palpable lymphadenopathy or organomegaly. Complete blood count was only significant for normocytic normochromic anemia with hemoglobin of 11.5 g/dL (12–16 g/dL). Other tests including C-reactive protein, liver enzymes, creatinine, blood urea nitrogen, and lactate dehydrogenase were normal. Anemia workup was consistent with anemia of chronic disease. T1 MRI enhanced with Gadolinium on a coronal plane is shown (Figure 2) and depicts a heterogeneous enhancement of an infiltrative lesion located at the internal border of the right mandible associated with changes on the signal intensity of the adjacent bone marrow.  Figure 3 (axial T1) also demonstrates altered bone marrow signal intensity of the right mandible with disruption of the external bone cortex caused by infiltration. The soft tissue mass component is located at the buccal and sublingual spaces of the oral cavity. There were no cervical lymphadenopathies detected. Oral lesion biopsy revealed through microscopy extensive infiltration of the mucosa for noncohesive lymphoid cells with classic morphology of Reed-Sternberg (RS) cells in the typical background of small mature lymphocytes, eosinophils, and plasma cells (Figures 4(a) and 4(b)). The immunohistochemistry study of the RS population confirmed the expression of CD30 (Figure 4(c)) and CD15. Epstein-Barr virus was also detected (Figure 4(d)). The RS cells neither expressed CD45, CD20, EMA, ALK, nor T cell markers such as CD3, CD5, CD4, and CD8. Antiviral capsid antigen immunoglobulin (Ig) G against Epstein-Barr virus (EBV) was positive, antiviral capsid antigen IgM was negative. EBV DNA copy number was negative (normal range: <10 copies). Staging studies with chest, abdominal, and pelvis CT scan and bone marrow biopsy indicated no further extension; erythrocyte sedimentation rate was 34 mm/hr with an albumin level of 3.8 g/dL. Based on these, a diagnosis of classical Hodgkin lymphoma Cotswold stage IEA was made. He was started on ABVD chemotherapy (doxorubicin, bleomycin, vinblastine, and dacarbazine) with planned duration of two to four cycles before initiation of involved field radiotherapy.  Figure 5 (coronal STIR) shows the evolution of the lesion after two cycles of ABVD chemotherapy, with no soft tissue mass present at the moment. Residual changes in bone marrow signal intensity can be seen.

## 2. Discussion

From the subset of malignancies affecting the head and neck region, lymphomas are the second most frequent after carcinomas. From those lymphomas, 80% to 90% are Non-Hodgkin lymphomas and about 4% represent Hodgkin lymphoma (HL) [1, 2]. The incidence of HL is estimated to be 7400 new cases per year in the United States, accounting for approximately 30% of all lymphomas [3]. As well known, most of the HL involves the lymph nodes. However, under some circumstances, those tumors arise from tissues other than the lymph nodes. Trying to define certain anatomical area, the term Waldeyer ring is used to include the lymphoid tissues of the faucial tonsils, nasopharynx, base of tongue, and oropharynx and therefore is considered an extranodal but not an extralymphatic site [4]. HL cases arising from this tissue, although uncommon, are being well characterized [5]. Therefore, the term extranodal, extralymphatic lymphoma has been used to describe the uncommon form of lymphoid malignancy, in which there is neoplastic proliferation at sites other than the expected native lymph nodes and lymphoid tissue, respectively. Due to the difficulty in case definition, the frequency of this type of variation is not well established, nevertheless when sites rich in primary lymphoid tissue such as Waldeyer's ring and spleen are considered extranodal, extranodal lymphomas would represent 25–50% of all non-Hodgkin lymphomas and only 2–5% of classical Hodgkin lymphomas [6]. In the case herein reported, the lymphoma was localized in the mandible with extension to the buccal mucosa; both considered outside the boundaries of the Waldeyer's ring. 

Extranodal, extralymphatic lymphomas from the bone are uncommon, comprising only 8% of primary malignant bone tumors and they are mainly diffuse B-cell type non-Hodgkin lymphomas [7]. This is probably the third case reported of a primary extranodal and extralymphatic oral Hodgkin lymphoma of the mandible [8, 9]. There is no information on the progression of the first case [8], but the second case relapsed 17 months after diagnosis and involved field radiotherapy of the pleura, spleen, liver, and para-aortic and inguinal lymph nodes, dying 6 months later after five cycles of chemotherapy with MOPP regimen (nitrogen, mustard, vincristine, procarbazine, and prednisone) [9]. Since the applicability of International Prognostic Score is restricted to advanced stage disease [10], a recent study showed that the absence of tumor associated macrophages represented by the marker CD68, such in this case, is strongly correlated with 100% ten-year disease specific survival [11]. Our case was in complete remission 3 months into therapy. We are describing the course of this rare HL presentation and the favorable early response to standard chemotherapy. This displayed the wide clinical spectrum that this type of tumor may encompass. Remarkable still little is known about the survival, prognosis, and effectiveness of the chemotherapy for this rare variant.

## Figures and Tables

**Figure 5:**